# Deletion of NADPH oxidase 2 attenuates cisplatin-induced acute kidney injury through reducing ROS-induced proximal tubular cell injury and inflammation

**DOI:** 10.3389/fmed.2023.1097671

**Published:** 2023-03-13

**Authors:** Ho-Ching Chen, Hsin-Yu Hou, Junne-Ming Sung, Chi-Chang Shieh

**Affiliations:** ^1^Institute of Clinical Medicine, College of Medicine, National Cheng Kung University, Tainan, Taiwan; ^2^Renal Division, Department of Internal Medicine, Cishan Hospital, Ministry of Health and Welfare, Kaohsiung, Taiwan; ^3^Renal Division, Department of Internal Medicine, National Cheng Kung University Hospital, Tainan, Taiwan; ^4^Department of Pediatrics, National Cheng Kung University Hospital, Tainan, Taiwan

**Keywords:** NOX2, ROS, cisplatin-induced AKI, ICAM-1 NOX2 modulates cisplatin-induced nephrotoxicity, neutrophil infiltration

## Abstract

**Backgrounds:**

Cisplatin is a commonly used chemotherapeutic agent in cancer treatment. However, its high nephrotoxicity limits its therapeutic application and efficacy. Cisplatin induces nephrotoxicity mainly through oxidative stress and inflammation. Reactive oxygen species (ROS) in the kidneys mainly arise from nicotinamide adenine dinucleotide phosphate (NADPH) oxidases 2 (NOX2), which is highly upregulated during ischemia-reperfusion injury and diabetes mellitus. However, its role in cisplatin-induced acute kidney injury (AKI) remains unknown.

**Methods:**

A 8-10-week-old NOX2 gene-knockout and wild-type mice were injected with 25 mg/kg cisplatin intraperitoneally for experiments.

**Results:**

We investigated the role of NOX2 in cisplatin-induced AKI and found that NOX2-mediated ROS production is a key inflammatory mediator of proximal tubular cell injury in cisplatin-induced AKI. NOX2 gene-knockout alleviated cisplatin-induced renal function decline, tubular injury score, kidney injury molecule-1(Kim-1) expression, and interleukin (IL)-6 and IL-1α levels with a reduction of ROS production. Moreover, in cisplatin-induced AKI, intercellular adhesion molecule 1 (ICAM-1) and the chemoattractant CXC ligand 1 (CXCL1) were highly expressed in association with neutrophil infiltration, which were all attenuated by deletion of NOX2.

**Conclusion:**

These data indicate that NOX2 aggravates cisplatin nephrotoxicity by promoting ROS-mediated tissue injury and neutrophil infiltration. Thus, appropriate targeting of NOX2/ROS pathway may minimize the risk of cisplatin-induced kidney injury in patients receiving cancer therapy.

## 1. Introduction

Cisplatin is a common chemotherapy agent used to treat several kinds of cancers. However, dose-dependent renal toxicity has been observed in clinical use and results in renal dysfunction and acute tubular necrosis (1). Approximately 1/3 of patients developed acute nephrotoxicity after a single dose of cisplatin (2). Furthermore, repeated low-dose cisplatin administration may cause the acute kidney injury (AKI) to progress to chronic kidney disease with renal fibrosis (3). This potentially lethal toxicity limits the use of this agent in cancer patients. Therefore, it is essential to understand the pathogenesis of cisplatin-associated nephrotoxicity in order to develop preventive strategies.

The uptake of cisplatin in the kidneys is through the organic cation transporters (4). Overexpression of organic cation transporter 2 in human proximal tubular cells enhances cisplatin uptake and increases the risk of toxicity (5). Cisplatin uptake into renal tubular cells causes proximal tubular cell toxicity, mitochondrial dysfunction, DNA damage, and decreased antioxidant glutathione levels, leading to the accumulation of reactive oxygen species (ROS) (6, 7). ROS are important in maintaining normal kidney gluconeogenesis, electrolyte transport, glucose transport, and hemodynamics (8). However, excessive ROS may cause cellular damage in a self-enhancing loop, causing oxidative injuries, autophagy, necrosis, apoptosis, and inflammation (9–11). Furthermore, persistent oxidative stress contributes to kidney fibrosis and chronic kidney disease in repeated low-dose cisplatin exposure in cancer patients (12, 13). Several antioxidant compounds, including N-acetylcysteine, dimethyl sulfoxide, and heme oxygenase-1, have been reported to protect against cisplatin-induced AKI in *in vivo* models, by suppressing ROS accumulation in the tissue (14–16).

Nicotinamide adenine dinucleotide phosphate oxidases (NOXs) contribute significantly to intracellular ROS generation in different animal models of kidney injury (17, 18). However, the role of NOXs in cisplatin nephrotoxicity is still unclear. There are seven isoforms of the NOX family (NOX1–5, Duox1/2). Different NOX isoforms are regionally distributed along the nephron segments (19, 20). The classic NOX2 is found primarily in phagocytic cells but is also expressed in renal proximal tubular cells. NOX2 upregulation was found in different animal kidney injury models including Akita and streptozotocin-treated diabetic mice, hypertension, and ischemia–reperfusion AKI (21–24). These data suggested that NOX2 plays an important role in renal oxidative stress and renal injury in certain kidney diseases. However, the role of NOX2 mediated ROS generation in cisplatin-induced AKI is not fully elucidated. In this study, we hypothesize that cisplatin nephrotoxicity is induced through NOX2-mediated mechanisms. Here we used a mouse model to show the role of NOX2 and potentially NOX2-produced ROS in proximal tubular cell injury and renal inflammation after cisplatin treatment.

## 2. Materials and methods

### 2.1. Mice

Mice deficient in Cybb, the gene encoding the gp91phox subunit of NOX2, (B6.129S-Cybbtm1Din/J, No. 002365) were purchased from Jackson Laboratory. C57BL/6 mice purchased from National Laboratory Animal Center were used as WT mice. All mice have been routinely backcrossed to C57BL/6 background, undergone genome-wide genotyping to confirm the genetic background, and housed in the animal facility of the Laboratory Animal Center at National Cheng Kung University. All mice were housed at the National Cheng Kung University Laboratory Animal Center and maintained under a temperature-controlled, 12 h light/dark cycle and specific pathogen-free conditions. We injected 25 mg/kg of cisplatin (CDDP) intraperitoneally into 8–10-week-old male mice, which were euthanized at 0 and 3 days after injection. Four to five mice were included in each group, and each experiment was repeated 2–3 times. All experimentation protocols were approved by the Institutional Animal Care and Use Committee (IACUC), at National Cheng Kung University.

### 2.2. Western blotting

Mouse renal cortices were lysed using 1× phosphate-buffered saline containing a protease inhibitor cocktail (Sigma-Aldrich, St. Louis, MO, United States). Protein concentrations were determined using a bicinchoninic acid protein assay kit (Thermo Fisher Scientific, Waltham, MA, United States). Denatured proteins were separated through sodium dodecyl sulfate-polyacrylamide gel electrophoresis and then transferred to polyvinylidene fluoride membranes, blocked with 5% non-fat milk in Tris-buffered saline/Tween buffer at 4°C overnight, and incubated with the following primary antibodies: rabbit anti-mouse NOX1 (GeneTex, Irvine, CA, United States), mouse anti-mouse NOX2 (gp91phox) (BD Transduction Laboratories, Franklin Lakes, NJ, USA), rabbit anti-mouse NOX4 (GeneTex), and rabbit anti-mouse GAPDH (GeneTex) at 1:500, 1:8000, 1:2000, and 1:10,000 dilution, respectively. The membranes were washed with Tris-buffered saline/Tween buffer and incubated with the appropriate horseradish peroxidase-conjugated secondary antibodies. Relative intensities of the proteins band were quantified using ImageJ version 1.49 (U.S. National Institutes of Health, Bethesda, MD, United States).

### 2.3. Immunofluorescence staining

Mouse kidneys were fixed, embedded in paraffin, and cut into 3-μM-thick sections with a microtome RM2235 and incubated with blocking solution using Novolink polymer detection system kit (Leica Biosystems, Wetzlar, Germany). The cortical sections of the kidney were deparaffined and rehydrated before incubation with rabbit anti-mouse aquaporin 1 (1:250; Abcam, Cambridge, United Kingdom) and mouse anti-mouse gp91phox (1:125; BD Transduction Laboratories) antibodies. 488-conjugated goat anti-rabbit immunoglobulin G and DyLight 594-conjugated goat anti-mouse albumin were used as secondary antibodies at 1:200 dilution, respectively. Cell nuclei were counterstained with 4′,6-diamidino-2-phenylindole. Images were captured under a fluorescence microscope (Carl Zeiss, Jena, Germany) equipped with AxioVision version 4.9.1 (Carl Zeiss, Jena, Germany).

### 2.4. Glutathione fluorometric assay

The tissue levels of glutathione and glutathione disulfide were measured with a glutathione fluorometric assay kit (BioVision, San Francisco, CA, United States), per the manufacturer’s instructions. Fresh renal cortices were lysed with a glutathione assay buffer, and perchloric acid (6 N) was added to achieve a uniform emulsion; this was followed by centrifugation at 13,000× *g* for 2 min. In perchloric acid–preserved samples, we added potassium hydroxide (6 N), a glutathione quencher, and an OPA probe. Samples were measured on a fluorescence plate reader.

### 2.5. Blood urea nitrogen and creatinine measurement

The mouse blood was allowed to stand for 1 h at room temperature and was then centrifuged at 100× *g* for 30 min; the supernatant was collected for further analysis. Blood urea nitrogen (BUN) and creatinine levels were measured using a FUJI DRI-CHEM slide (FUJIFILM, Tokyo, Japan) and FUJI DRI-CHEM Analyzer (FUJIFILM).

### 2.6. Periodic acid–schiff staining

Sections were deparaffined, dehydrated, and stained using a periodic acid–schiff (PAS) staining kit (Abcam, Cambridge, United Kingdom), as described by the manufacturer. Tubular injury was scored by a blinded observer who examined cortical area of kidney tissue (× 200 magnification) of PAS-stained sections. Twenty images in each group were captured with a light microscope (Carl Zeiss). Histopathological changes were evaluated by the percentage of damaged renal tubules, as indicated by tubular cell necrosis, tubular dilatation, loss of the brush border, and cast formation. Tubular injury was quantified based on a 5-point scoring system, with 0, 1, 2, 3, 4, and 5 point(s) corresponding to 0, <10, 11–25%, 26–50%, 51–75%, and ≥ 76% tissue damage, respectively.

### 2.7. Immunohistochemical staining

The cortical area of kidney tissue was cut into 3-μM-thick sections, as described earlier, which were blocked before incubation of goat anti-mouse anti-Kim1 (R&D Systems, Minneapolis, MN) or mouse anti-mouse anti-ICAM1 (Abcam, Cambridge, United Kingdom) at 1:800 and 1:2000 dilution, respectively. Immunohistochemical staining was performed using a Novolink polymer detection system (Leica Biosystems, Wetzlar, Germany), as described by the manufacturer. The sections were counterstained with hematoxylin. Images were captured with a light microscope (Carl Zeiss) equipped with AxioVision version 4.9.1 at 400× magnification. The respective signal area and density were measured using ImageJ (version 1.49; U.S. National Institutes of Health, Bethesda, MD, United States).

### 2.8. Luminex assay

The supernatants of the renal cortex were assayed for the following proteins, cytokines, and chemokines: Kim-1, vascular endothelial growth factor (VEGF), soluble intercellular adhesion molecule (sICAM), sP-selectin, interleukin (IL)-4, IL-5, IL-6, IL-10, IL-17A, monocyte chemoattractant protein 1 (MCP-1), GM-CSF, IL-1α, MIP-2 (CXC ligand 2 [CXCL2]), and KC (CXC ligand 1 [CXCL1]). These assays were performed in 96-well plates, and compounds were detected with a MILLIPLEX MAP kit (Merck, Darmstadt, Germany), as described by the manufacturer.

### 2.9. Flow cytometry

The kidneys were cut into small pieces, digested in a complete medium containing collagenase D (1,400 μg/mL) and DNase I (100 μg/mL) for 45 min at 37°C, and then passed through a 70-μM strainer. For leukocyte purification, the samples were subjected to Percoll gradient centrifugation. Single-cell suspensions were blocked with Fc block (anti-CD16/32, catalog no. 553142; BioLegend, San Diego, CA, United States) and stained with fluorescently conjugated antibodies against CD45 (30-F11), CD11b (M1/70), CD11c (HL3), Ly6G (1A8), and F4/80 (BM8). All antibodies were obtained from BioLegend and BD Transduction Laboratories (Franklin Lakes, NJ, United States). A single-stained control for each fluorochrome was made for compensation for each experiment. Labeled cells were detected by flow cytometry with FACS Calibur (BD Biosciences) or FACS Canto II instruments (BD Biosciences) and analyzed with the FlowJo software program (FlowJo, LLC, Ashland, OR, United States).

### 2.10. Cell culture

Human kidney tubular epithelial cells (HK2) (BCRC number: 60097) derived from normal kidney transfected with human papilloma virus 16 were obtained from Bioresource Collection and Research Center, Taiwan, and cultured in DMEM/F12 (Gibco) containing 10% FBS. The cell culture was kept at 37°C and 5% CO_2_ condition.

### 2.11. Treatment of HK2

HK2 (1 × 10^6^ cells/well for 6-well plates) were plated overnight in a complete medium. Cells were treated with or without 5 μM diphenyleneiodonium chloride (DPI), a potent irreversible ROS inhibitor, at 37°C for 30 min then incubated with 2 μM cisplatin (CDDP) for 24 h. The levels of ROS were measured using a fluorescent dye 2′, 7′-dichlorofluorescein diacetate (DCFDA) cellular ROS assay kit (Abcam). Cells were harvested and stained with DCFDA at 37°C for 30 min then analyzed with flow cytometry.

### 2.12. Statistical analysis

All quantitative data are expressed using the mean ± standard error of the mean values. Data were evaluated with a Student’s *t* test between two groups or a one-way or two-way analysis of variance when comparing more than three groups. SPSS (version 23; IBM Corporation, Armonk, NY, United States) was used for all statistical analyzes, and *p* < 0.05 was statistically significant. The statistically differences between groups are indicated with *p < 0.05, **p < 0.01, ns: no significance.

## 3. Results

### 3.1. Increased expression of NOX2 in cisplatin-induced AKI

**Figure 1:**
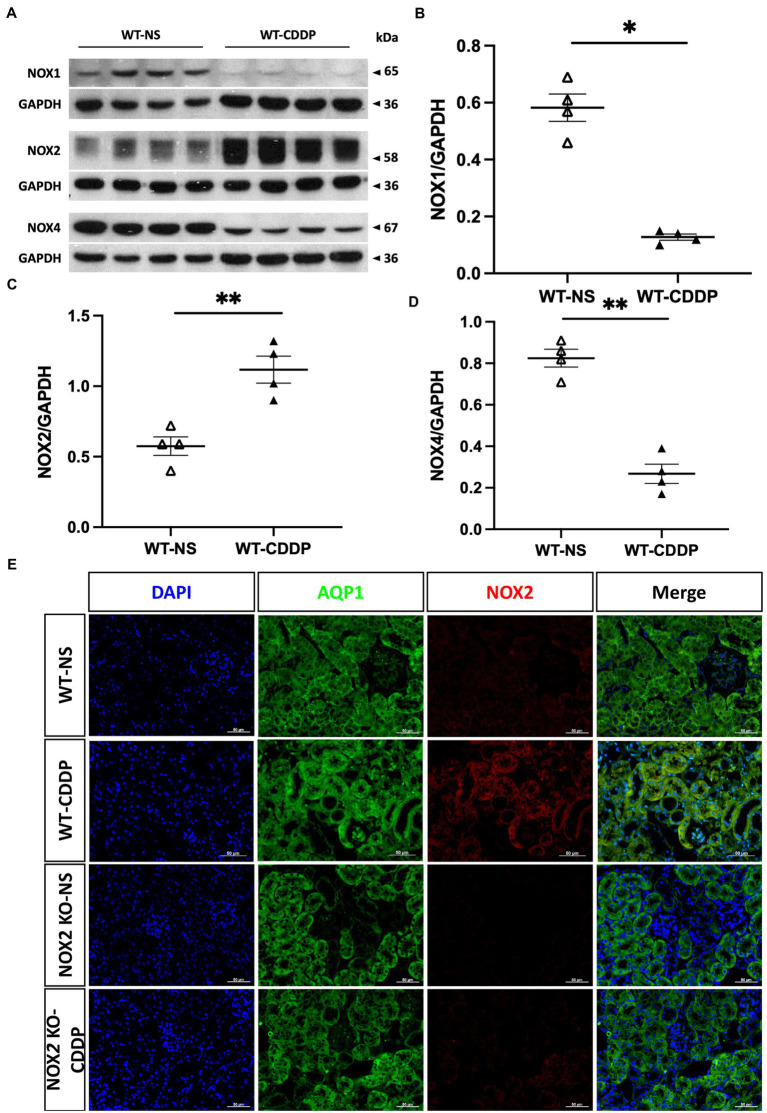
Increased NOX2 expression in cisplatin-induced AKI. Three days after cisplatin injection, western blot analysis was used to examine the main NOX family expression in cisplatin-induced AKI. **(A)** Western blots of NOX1, NOX2, and NOX4. **(B–D)** Quantitative analysis of NOX2 indicated overexpression compared to WT mice. However, NOX1 and NOX4 decreased after cisplatin injury. **(E)** Immunofluorescence staining suggests NOX2 expression in the epithelial cells along the renal proximal tubule in cisplatin-induced AKI. NOX2-KO mice did not show NOX2 expression in the normal saline and cisplatin groups. Data represent the mean ± standard error of four mice per group. Scale bar = 50 μM. WT-NS, wild type-normal saline; WT-CDDP, wild type-cisplatin; AQP1: aquaporin 1.

**Figure 2:**
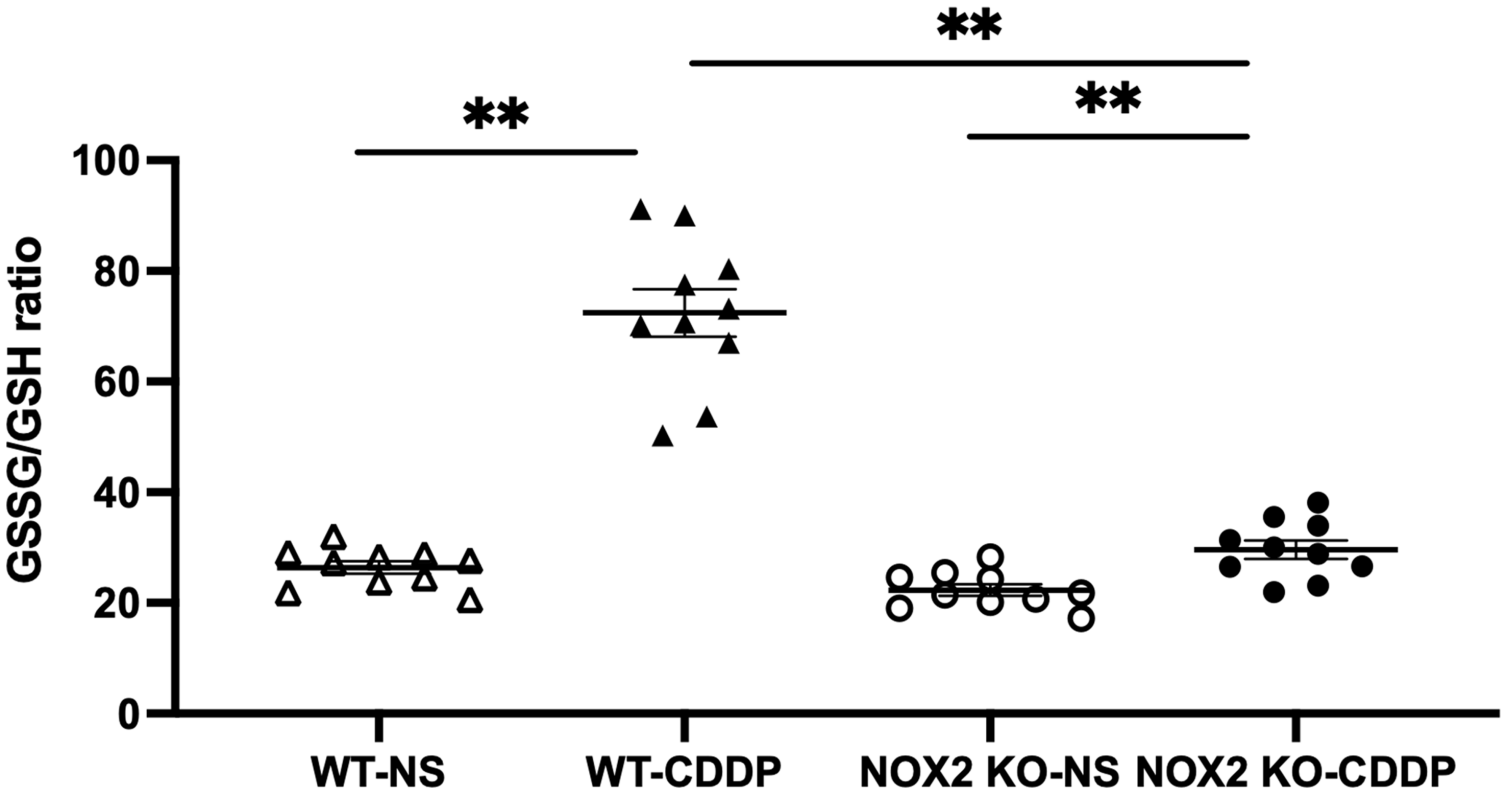
NOX2-KO mice markedly attenuate the cisplatin-induced ROS increase in WT mice. The glutathione disulfide/glutathione ratio, an indicator of oxidative stress, was increased 3 days after cisplatin injection in the WT group. In contrast, ROS were a significantly blunt in the NOX2-KO CDDP group compared with the WT-CDDP group. Data represent the mean ± standard error of five mice per group. The experiment was repeated twice with similar results.

Three days after cisplatin injection, the NOX2 protein level significantly increased in cisplatin-induced AKI (Figures 1A,C), whereas the NOX1 and NOX4 protein levels did not increase (Figures 1B,D). We verified the location of NOX2 expression in the renal tubular cells after cisplatin induction with immunofluorescence staining (Figure 1E). In the normal condition, no NOX2 expression was observed in the wild-type (WT) normal saline (NS) group. NOX2-knockout (KO) mice did not exhibit NOX2 expression in either the cisplatin or NS group. ROS play important role in cisplatin kidney toxicity; thus, we performed a glutathione fluorometric assay to determine the amount of ROS production. We observed that oxidative stress was significantly increased in the WT cisplatin-treated group compared to the NOX2-KO group (58% increase), indicating that cisplatin-related ROS generation in kidney tissues may be largely through NOX2 (Figure 2). We also confirmed that the NOX inhibitor DPI was significantly reduced ROS production in cisplatin-treated human kidney tubular epithelial cells injury *in vitro* (Supplementary Figure S1). Both genetic and pharmacological inhibition of NOX2-derived ROS in cisplatin-induced AKI could be a potential therapeutic target.

### 3.2. NOX2 deficiency protected against cisplatin-induced AKI *in vivo*

**Figure 3:**
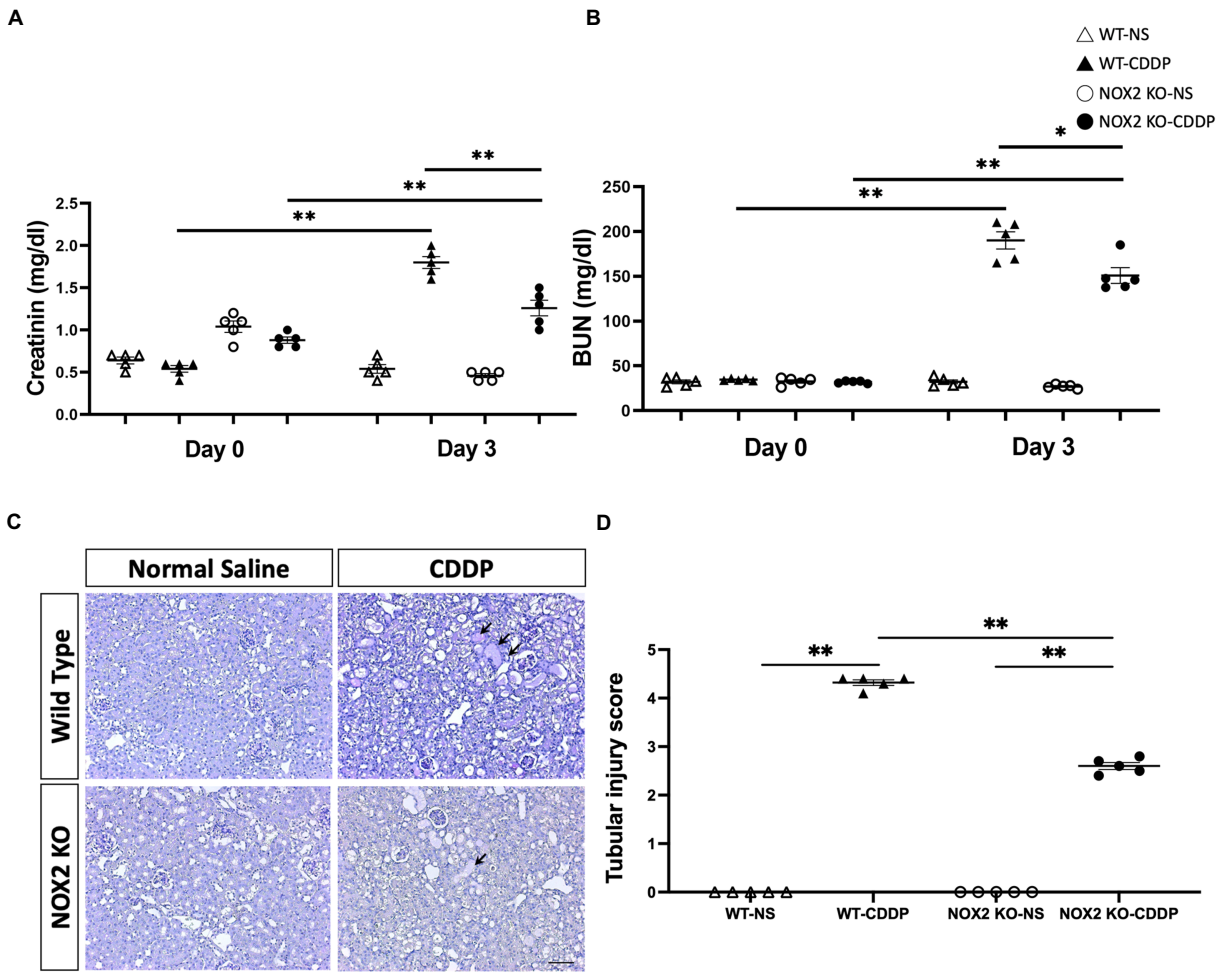
Mice with NOX2 deletion had milder cisplatin-induced AKI. **(A)** Serum creatinine levels indicated that NOX2-KO suppressed the cisplatin-mediated decrease in renal function. **(B)** BUN levels had a lower trend in NOX2 KO-CDDP mice compared with WT CDDP mice. **(C,D)** PAS staining of renal tubules revealed cisplatin-induced cast formation, tubular dilatation (black arrow), and tubular epithelial cell injury in WT CDDP mice. Quantitative analysis of PAS staining indicated NOX2 deletion suppressed the tubular injury score 3 days after cisplatin injection. Data represent the mean ± standard error of five mice per group. Scale bar = 100 μM.

**Figure 4:**
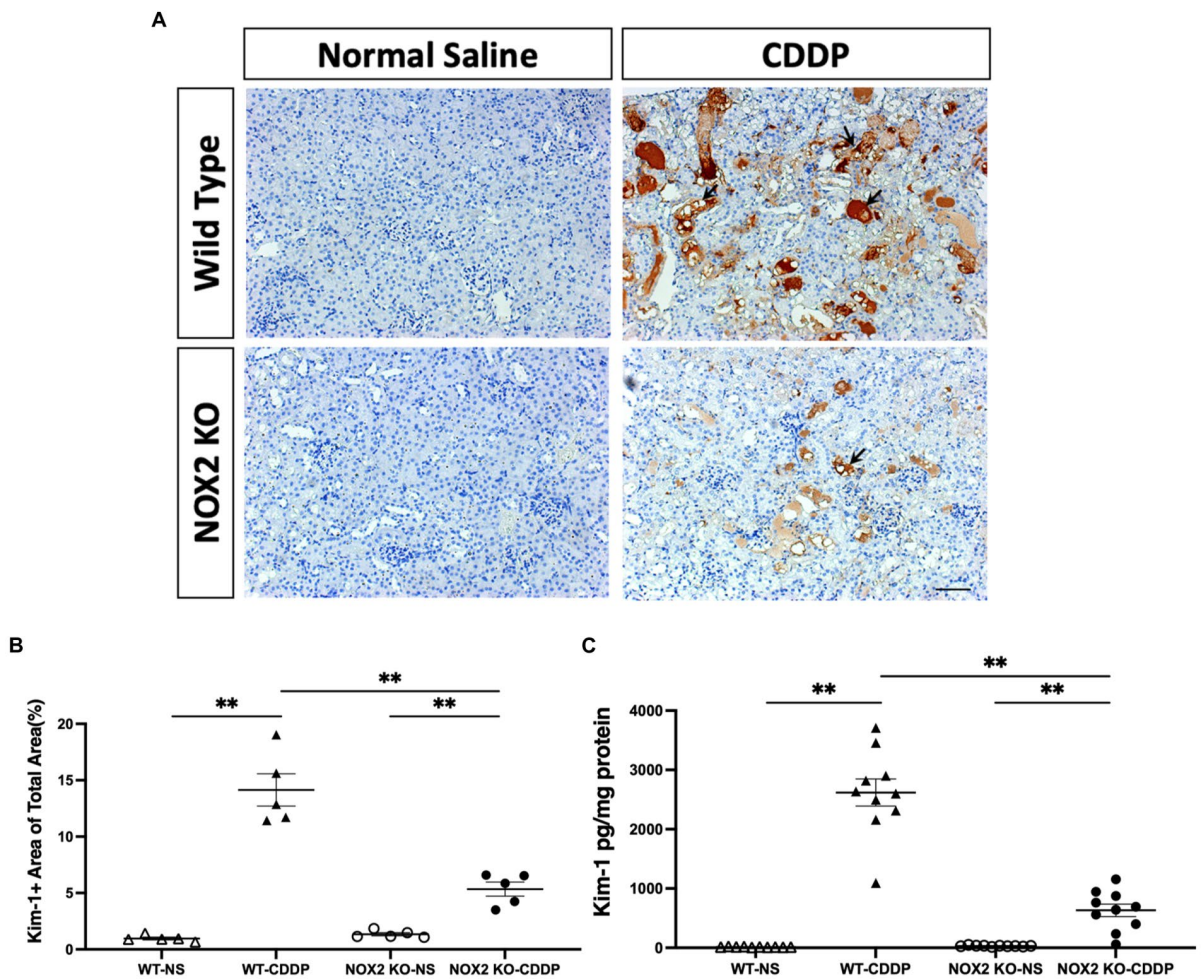
Mice with NOX2 deletion had decreased Kim-1 levels in cisplatin-induced AKI. **(A,B)** Kim-1, a biomarker of renal tubular injury, was overexpressed and accumulated in the WT CDDP group’s peritubular area (black arrow). Immunohistochemical analysis indicated that the area of Kim-1 was greater after cisplatin and reduced in the NOX2 KO mice. **(C)** NOX2 deletion reduced Kim-1 protein levels in cisplatin-induced AKI, as indicated by the Luminex assay. Data represent the mean ± standard error of five mice per group. Scale bar = 100 μM. The experiment was repeated twice with similar results.

We observed that the knockout of NOX2 attenuated cisplatin-induced renal damage with a significantly decrease of serum creatinine level (30% decrease) (Figure 3A) and a lower level of BUN (Figure 3B) 3 days after cisplatin injection. PAS staining demonstrated that kidneys in the WT-CDDP group had a more severe tubular injury with cast formation, tubular dilation, and necrosis. In contrast, a deficiency of NOX2 attenuated the degree of tubular injury score (Figures 3C,D). In addition, kidney injury molecule-1 (Kim-1), an early AKI biomarker, is a sensitive indicator of proximal tubular injury. Immunohistochemistry and quantitative data demonstrated that Kim-1 was significantly lower in the NOX2-deficient CDDP group (Figures 4A,B) This was further confirmed through a Luminex assay, which revealed that the disruption of NOX2 significantly reduced the protein level of Kim-1 (78% decrease) after cisplatin injection (Figure 4C). These results further confirmed that NOX2 KO decreases the severity of cisplatin-induced proximal tubular cell injury and AKI.

### 3.3. NOX2 deletion attenuated cisplatin-induced pro-inflammatory cytokines expression

**Figure 5:**
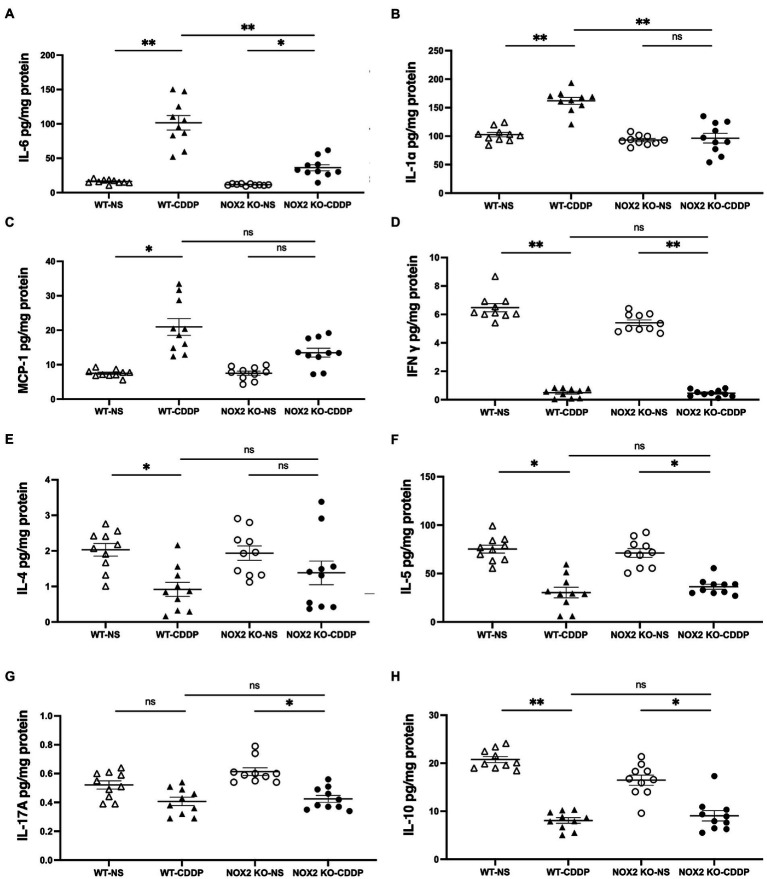
Cytokine expression with Luminex assay in cisplatin-induced AKI in the WT and NOX2-KO groups. **(A,B)** IL-6 and IL-1α proinflammatory factors were significantly reduced in the NOX2 KO-CDDP group compared to the WT-CDDP group. **(C–G)** MCP-1 and T-helper lymphocyte cytokines such as IFN-γ, IL-4, IL-5, and IL-17A did not show significant changes between the WT-CDDP and NOX2 KO-CDDP groups. **(H)** IL-10 was significantly reduced in the WT-CDDP group compared with the WT-NS group. IL-10 was mildly recovered in the NOX2 KO-CDDP group compared with the WT-CDDP group. Data represent the mean ± standard error of five mice per group. The experiment was repeated twice with similar results.

Cisplatin induces the expression of various cytokines in renal tissue, which was associated with further renal damage (25). We observed that the pro-inflammatory factors IL-6 and IL-1α were significantly reduced (by 64 and 47%, respectively) in the NOX2-KO CDDP group compared to the WT CDDP group (Figures 5A,B). IL-6 and IL-1α are important mediators of the acute-phase response and are released in the event of sepsis, ischemic injury, or toxicity. IL-6 is secreted by macrophages, and IL-1α is produced mainly by activated neutrophils as well as by macrophages, epithelial cells, and endothelial cells. However, MCP-1 exhibits a chemotactic activity for monocytes, which did not exhibit an obvious change in either the WT CDDP or NOX2-KO CDDP group (Figure 5C). We did not find that Th1, Th2, or Th17 cytokines exhibited any difference in the NOX2-KO CDDP group (Figures 5D–G). IL-10 production by dendritic cells was found to have a protective effect on cisplatin-induced AKI (26). Our data also indicated a mild elevation of IL-10 in the NOX2-KO CDDP group compared to the WT CDDP group (Figure 5H). Thus, these data indicate that NOX2 deletion prevented the cisplatin-induced change in cytokines.

### 3.4. Reduction of neutrophil infiltration in attenuated cisplatin-induced AKI in NOX2 deficient mice

**Figure 6:**
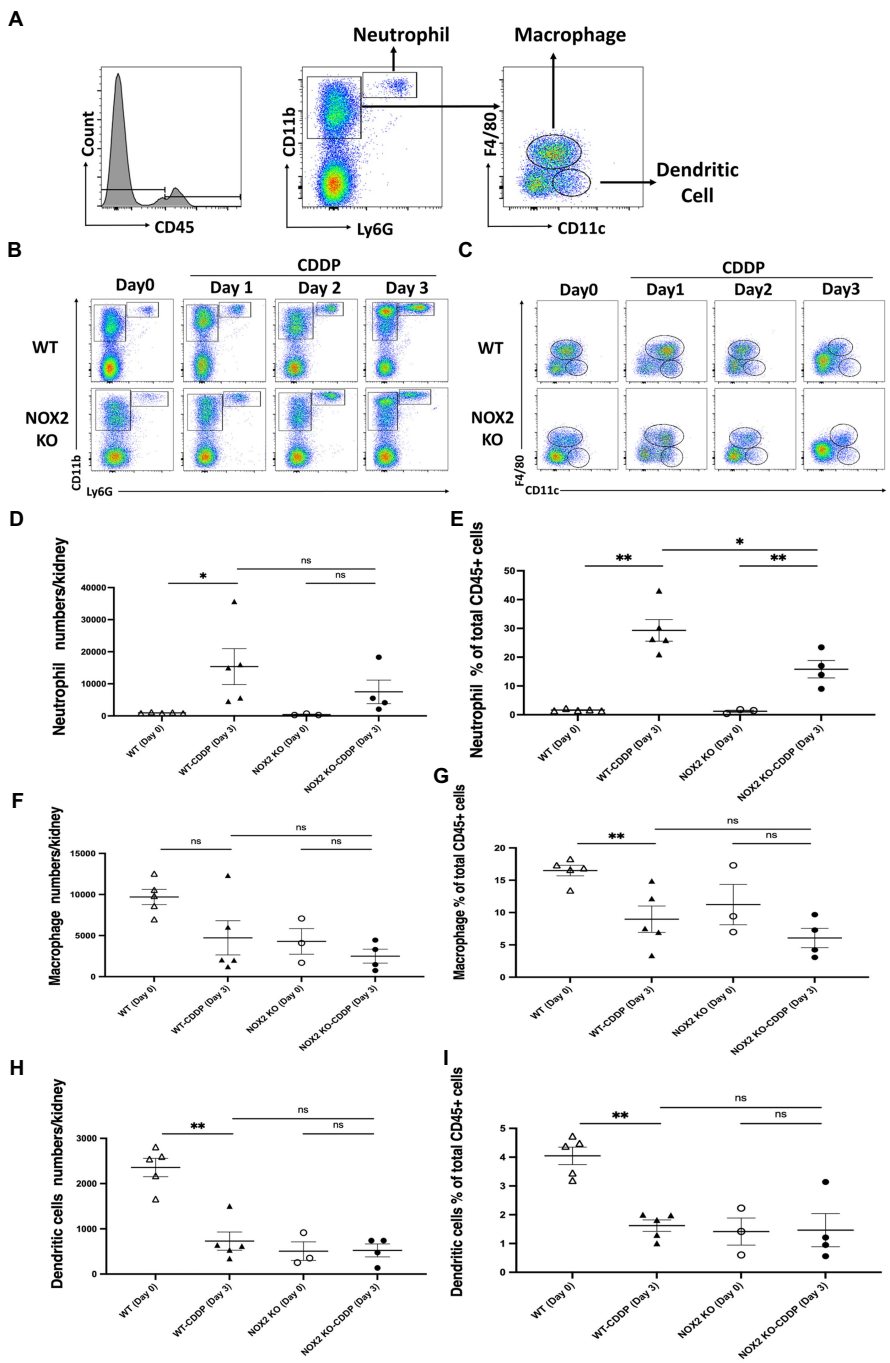
Mice with NOX2 deletion had reduced neutrophil infiltration in cisplatin-induced AKI. **(A)** Whole kidneys were homogenized into a single-cell suspension for flow cytometric analysis of immune cells. Hierarchical gating was performed to identify Ly6G^+^, CD11b^+^ neutrophils; F4/80^+^, CD11C^−^ macrophages; and F4/80^−^, CD11c^+^ dendritic cells. **(B,D,E)** Neutrophil infiltration was increased on day 3 after cisplatin injection in both WT and NOX2 KO groups. However, the neutrophil percentage of total CD45^+^ cells was significantly reduced in the NOX2-KO CDDP group on day 3 compared to that of the WT CDDP group. **(C,F–I)** The percentages of macrophage and dendritic cells among total CD45^+^ cells were significantly reduced in the WT CDDP group on day 3 compared to the WT on day 0. However, the numbers and percentages of total CD45^+^ macrophage and dendritic cells were not significantly different between the WT CDDP and NOX2-KO CDDP groups on day 3. Data represent the mean ± standard error of 3–5 mice per group.

Inflammatory cells of the immune system, such as neutrophils, macrophages, and dendritic cells, infiltrate the kidney tissue and affect the development of cisplatin-induced AKI. We further assessed the composition of immune cells in the kidneys to elucidate the underlying mechanism by which NOX2 deficiency alleviates AKI. The gating strategy included Ly6G^+^, CD11b^+^ neutrophils, F4/80^+^, CD11C^−^ macrophages; and F4/80^−^, CD11c^+^ dendritic cells (Figure 6A). Flow cytometry revealed that the neutrophil count in the kidneys and the percentage of total CD45^+^ cells were significantly increased on days 3 after cisplatin injection in the WT group. However, NOX2 deficiency reduced neutrophil infiltration (by 38%) in the kidneys (Figures 6B,D,E). No differences were found in cell numbers or percentages of kidney macrophages and dendritic cells in cisplatin-treated WT mice and NOX2-KO mice (Figures 6C,F–I). Taken together, these data suggest that NOX2 deletion reduces neutrophil infiltration and attenuates cisplatin-induced AKI, consistent with the decreased levels of pro-inflammatory cytokines IL-6 and IL-1α (Figures 5A,B). Since neutrophils may constitute an important innate immune response in cisplatin-induced nephrotoxicity (27), our data suggest NOX2-induced neutrophil infiltration and inflammation may play a role in cisplatin-induced AKI.

### 3.5. Significantly decreased ICAM-1 and CXCL1 expression in cisplatin-induced AKI in NOX2-KO mice

**Figure 7:**
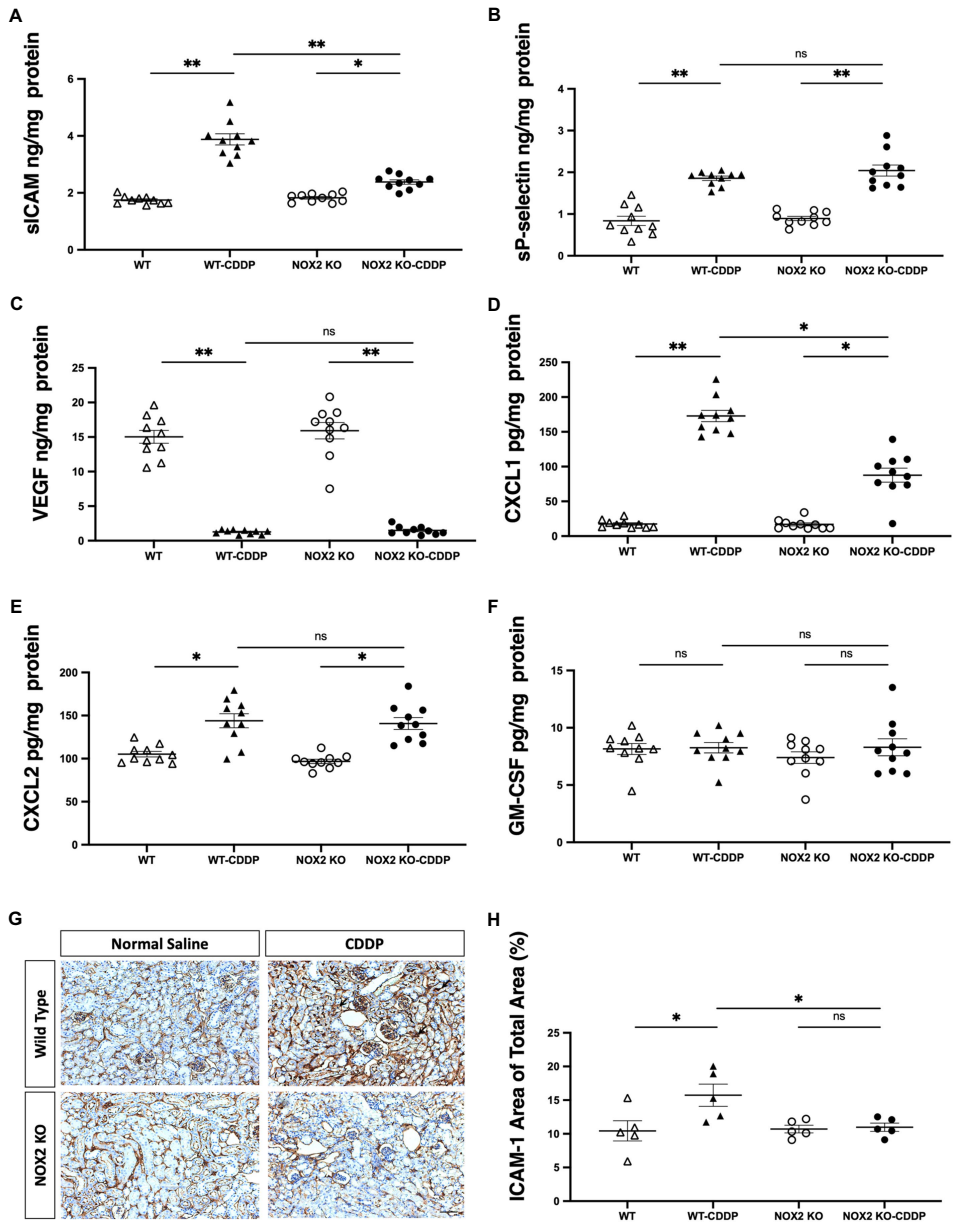
Cisplatin-induced increase in ICAM-1 and CXCL1 was reduced in NOX2-KO mice. **(A,G,H)** sICAM expression with Luminex assay was decreased in the NOX2-KO CDDP group compared to the WT CDDP group on day 3, and the area of ICAM-1 labeling (black arrow) through immunohistochemical staining was increased in WT-CDDP but not in NOX2 KO-CDDP mice. **(B,C,F)** sP-selectin, VEGF, and GM-CSF levels with Luminex assay were not significantly different between the WT CDDP and NOX2-KO CDDP groups on day 3. **(D,E)** The level of chemokine CXCL1 was significantly lower in the NOX2-KO CDDP group on day 3. However, another chemokine, CXCL2, showed no change between the WT CDDP and NOX2-KO groups. Data represent the mean ± standard error of five mice per group. Scale bar = 100 μM. The experiment was repeated twice with similar results.

To study the mechanism of reduced neutrophil infiltration in the kidneys observed in NOX2-KO mice treated with cisplatin, we analyzed the neutrophil recruitment cascade. Chemoattractants, such as CXCL1 and CXCL2 are key players in neutrophil activation. Activated neutrophils interact with endothelial cells, which slows them down. This is mediated by binding molecules, such as ICAM-1, P-selectin, and E-selectin. We observed that sICAM and CXCL1 were greatly reduced (by 32 and 51%, respectively) in the NOX2-KO CDDP group (Figures 7A,D). Immunohistochemical staining also revealed the area of ICAM-1 labeling was significantly increased in WT-CDDP group but not in NOX2-KO CDDP mice (Figures 7G,H). However, compared to WT CDDP mice, no significant differences in P-selectin and CXCL2 were noted in NOX2-KO CDDP mice (Figures 7B,E). We also assessed VEGF, which regulates vessel growth after AKI. VEGF decreased markedly after cisplatin in both WT and NOX2-KO mice. (Figure 7C). GM-CSF was previously demonstrated to be expressed in tubular cells after renal injury to promote macrophage alternative activation (28). However, GM-CSF expression did not differ among the four groups (Figure 7F). These results demonstrate that NOX2-KO mice exhibited significantly decreased ICAM-1 and CXCL1 expression consistent with lower neutrophil infiltration in AKI.

## 4. Discussion

In this study, we demonstrated the hitherto unknown importance of NOX2-mediated ROS production in cisplatin-induced AKI. ROS play an important role in cell signaling, oxygen sensing, apoptosis, gene expression, and cell defense (29–33). The major source of renal ROS generation in cisplatin-induced AKI is NADPH oxidases (34). NOX2-mediated ROS play an important pathogenetic role in different kidney diseases. Angiotensin-converting enzyme-2 attenuates diabetic kidney injury in the Akita mouse model in association with decreased NOX2 activity (20). It has been shown that NOX2 modulates ischemia–reperfusion injury in delayed graft function and that its absence is associated with reduced inflammation and fibrosis (35). Our findings also demonstrated that the deletion of NOX2 was associated with reduced ROS level and proinflammatory cytokines in cisplatin-induced AKI. Moreover, the lowered endothelial adhesion molecule ICAM-1 and chemokine CXCL1 may contribute to the reduction in neutrophil infiltration in NOX2 deficient mice in cisplatin-induced renal damage.

The pathogenesis of cisplatin-induced AKI is complex and involves both the innate and adaptive immune systems (25, 36). Cisplatin is cleared from the body by the kidney mainly by glomerular filtration. The renal proximal tubular cells are exposed to cisplatin toxicity through tubular secretion and uptake. The renal tubules may get injured by several mechanisms including apoptosis, DNA damage, direct toxicity, and inflammation (11). Although the secretion of IL-1, IL-6, and IL-18 in cisplatin-induced kidney injury has been well documented, the inhibition of these cytokines does not protect against cisplatin-induced AKI (25). Our results revealed that NOX2 KO prevents the increase in pro-inflammatory cytokines including IL-6 and IL-1α induced by cisplatin (Figures 5A,B). The levels of other cytokines, namely, interferon-γ, IL-4, IL-5, and IL-17A, did not significantly change in our study (Figures 5D–G). IL-10, an anti-inflammatory cytokine, was significantly reduced in WT cisplatin-induced AKI in our study. NOX2-KO mice with cisplatin injury exhibited a trend of IL-10 recovery (in terms of level) compared to WT mice (Figure 5H). Put together, these data indicate that NOX2 in renal epithelial cells increased ROS generation to promote pro-inflammatory cytokines and reduce anti-inflammatory cytokines.

After the initial tubular injury, there is an immediate inflammatory response, resulting in increased renal vascular endothelium permeability and the release of pro-inflammatory cytokines and chemokines. Numerous inflammatory cells, including neutrophils, macrophages, dendritic cells, and lymphocytes, infiltrate the kidneys and are important in the progression of cisplatin-induced AKI (25, 37–40). Therefore, we investigated the distribution of immune cells after cisplatin-induced AKI. Notably, NOX2 deletion reduced the cisplatin-induced increase in neutrophil infiltration (Figure 6E; Supplementary Figure S2). Activated neutrophils are important first responders in the innate immune system. They may cause damage in renal tubular cells by establishing an inflammatory cascade in the tissue. There are some controversial studies on the pathogenetic role of neutrophils in cisplatin-induced AKI. Depletion of neutrophils using the anti–GR-1 antibody or anti-Ly6G antibody did not affect renal cisplatin-induced injury (25, 41). However, a recent study revealed that blocking neutrophil infiltration by inhibiting the leukotriene B4 axis has protective effects against cisplatin-induced AKI (27). Our study suggested that the inhibition of NOX2-mediated ROS generation may attenuate kidney neutrophil infiltration and protects against cisplatin-induced AKI.

Neutrophil transmigration requires complex steps with a consequence of adhesive interaction with endothelial cells. Neutrophil recruitment depends on E-selectin, P-selectin, and ICAM-1 in the peritubular capillary (42). Increased vascular permeability and renal cytokines expression promote neutrophil transmigration. It has been shown that using anti-ICAM-1 antibody could significantly reduce neutrophil infiltration (43). Several research showed that IL-6 plays an important role in local inflammation and augmentation of ICAM-1 in vascular endothelial cells amplifying neutrophil recruitment (44–46). Chemokines CXCL1 and CXCL2 act sequentially to guide neutrophil crawling and transmigration during inflammation (47). Mice deficient in the CXCL1 receptor had a less renal tubular injury in cisplatin-induced AKI (48). We found that ICAM-1 and CXCL1 expression significantly decreased in cisplatin-induced AKI in NOX2-KO mice (Figures 7D,H), implicating the essential role of neutrophil recruitment in this ROS-dependent regulation.

Our study is the first to show that NOX2 was highly expressed in cisplatin-induced AKI. We hence used NOX2-KO mice to elucidate the importance of NOX2-derived ROS in mediating cisplatin-induced kidney injury. NOX2-KO mice exhibited attenuated renal damage and improvements in renal function and tubular injury as well as ROS reduction (Figures 2, 3). Moreover, the early AKI biomarker Kim-1 expressed by proximal tubular epithelial cells after injury was also significantly reduced in NOX2-KO mice (Figure 4). Our data indicate that NOX2 is pathogenic in cisplatin-induced AKI because the upregulation of NOX2 in renal tubular epithelial cells results in excessive ROS production, increases renal injury, and enhances neutrophil infiltration through chemokine CXCL1 and vascular endothelial ICAM-1 overexpression. Of the NOX isoforms, NOX4 is also upregulated in the various renal pathological process (49, 50). A recent study indicated that NOX4 aggravates cisplatin-induced AKI *via* programmed cell death and inflammation (51). The western blotting data in that study, like our results (Figures 1A,C), also showed NOX2 upregulation in cisplatin-treated HK2 cells. According to previous studies in different disease models, NOX isoform expression was also different. NOX4 expression was not upregulated in response to cisplatin treatment in our experiments may be due to different mouse strains and cisplatin dosages used in the experiments. Excessive ROS from NOX2 maybe have another pathway to inhibit the expression of NOX1 and NOX4.

Our study has some limitations. The cell types that generate nephrotoxic NOX2-derived ROS were not identified in the present study. We found NOX inhibitor indeed reduced the production of intracellular ROS in cisplatin-treated human kidney tubular epithelial cells (Supplementary Figure S1). NOX2-derived ROS are critical in regulating the function and gene expression of neutrophils (31–33). NOX2-deficient neutrophils have higher pro-inflammatory activities; this explains the more severe arthritis seen in the absence of NOX2 in mice (29, 30). However, cisplatin-induced AKI may have a different tissue microenvironment as compared with that of arthritis-inflamed joints. Further studies about the possible role of NOX2 and NOX2-derived ROS from the renal infiltrated neutrophil in cisplatin-induced AKI are needed.

**Figure 8:**
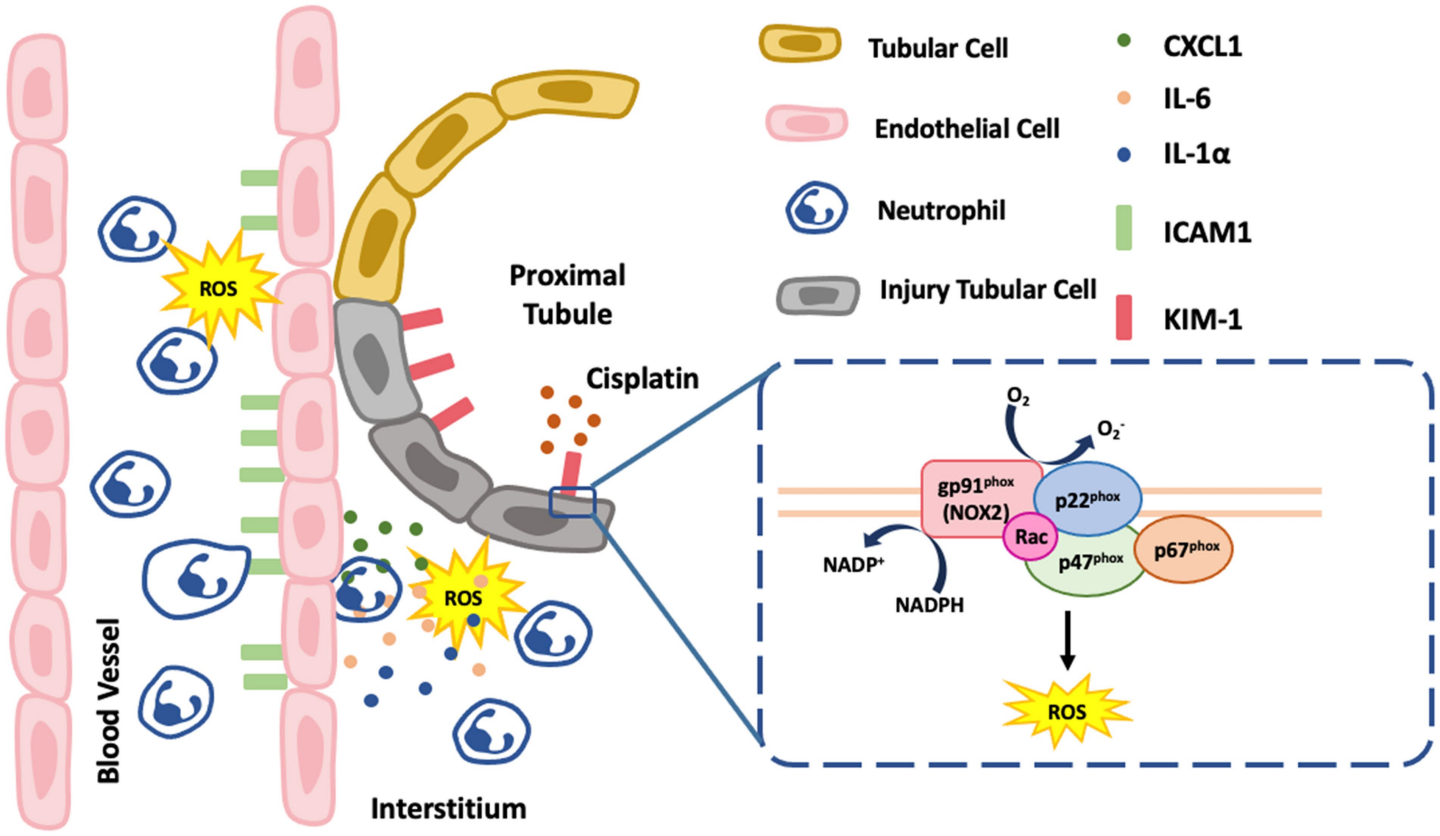
Schematic summarizing the mechanism of NOX2-induced ROS in the renal proximal tubular cells. NOX2 contributes to cisplatin-induced AKI and involves an increase Kim-1 and proinflammatory cytokines, IL6 and IL-1α which also increase neutrophil infiltration with higher endothelial adhesion molecule ICAM-1 and chemokine CXCL1. The box in the figure is NOX2 which has multiple membrane-bound subunits of NADPH oxidase including gp91^phox^, p22^phox^, p67^phox^, p47^phox^, and Rac.

In summary, ROS derived from NOX2 in renal tubular epithelial cells play a critical role in the pathophysiology of cisplatin-induced AKI (Figure 8); specifically, these ROS enhance proximal tubular cell injury and severity of renal function decline in cisplatin-induced AKI. Neutrophil infiltration, which may presumably be through NOX2-derived ROS, were associated with the generation of the chemokine CXCL1 and the vascular endothelial adhesion molecule ICAM-1 by pro-inflammatory cytokines stimulation such as IL-6 and IL-1α. NOX2 KO *in vivo* significantly attenuated cisplatin-induced AKI and inflammation, indicating that precise control NOX2/ ROS pathway may be a novel therapeutic strategy against cisplatin-induced AKI.

## Data availability statement

The original contributions presented in the study are included in the article/Supplementary material, further inquiries can be directed to the corresponding authors.

## Ethics statement

The animal study was reviewed and approved by Institutional Animal Care and Use Committee (IACUC), at National Cheng Kung University.

## Author contributions

H-CC and H-YH performed the experiments, analyzed the data, and designed the study. H-CC wrote the manuscript. H-CC, H-YH, J-MS, and C-CS analyzed the data and contributed to the discussion. C-CS and J-MS reviewed and edited the manuscript.

## Funding

This work was supported by the Ministry of Science and Technology, Taiwan (MOST107-2314-B-650-009) and by grants from EDAH (EDAHP106031 and EDAHP108029).

## Conflict of interest

The authors declare that the research was conducted in the absence of any commercial or financial relationships that could be construed as a potential conflict of interest.

## Publisher’s note

All claims expressed in this article are solely those of the authors and do not necessarily represent those of their affiliated organizations, or those of the publisher, the editors and the reviewers. Any product that may be evaluated in this article, or claim that may be made by its manufacturer, is not guaranteed or endorsed by the publisher.

## Supplementary material

SUPPLEMENTARY FIGURE 1 Inhibition of ROS by DPI, NOX inhibitor, reduced cisplatin-treated human kidney tubular epithelial cells injury. ROS generation were a significant increase in cisplatin-treated HK2 cells which were inhibited by DPI. DCFDA is a fluorogenic dye that measures ROS activity within the cell which is detected by flow cytometry. Data represent the mean ± standard error of four data per group.

SUPPLEMENTARY FIGURE 2 Time course analysis neutrophil, macrophage, and dendritic cells from day 0 to day 3 of cisplatin injection in WT and NOX2 KO mice.
