# Supplementary figures and images for: Deletion of NADPH oxidase 2 attenuates cisplatin-induced acute kidney injury through reducing ROS-induced proximal tubular cell injury and inflammation
Source: Front Med (Lausanne). 2023 Mar 13;10:1097671. doi: 10.3389/fmed.2023.1097671 (PMC10040743; doi:10.3389/fmed.2023.1097671)

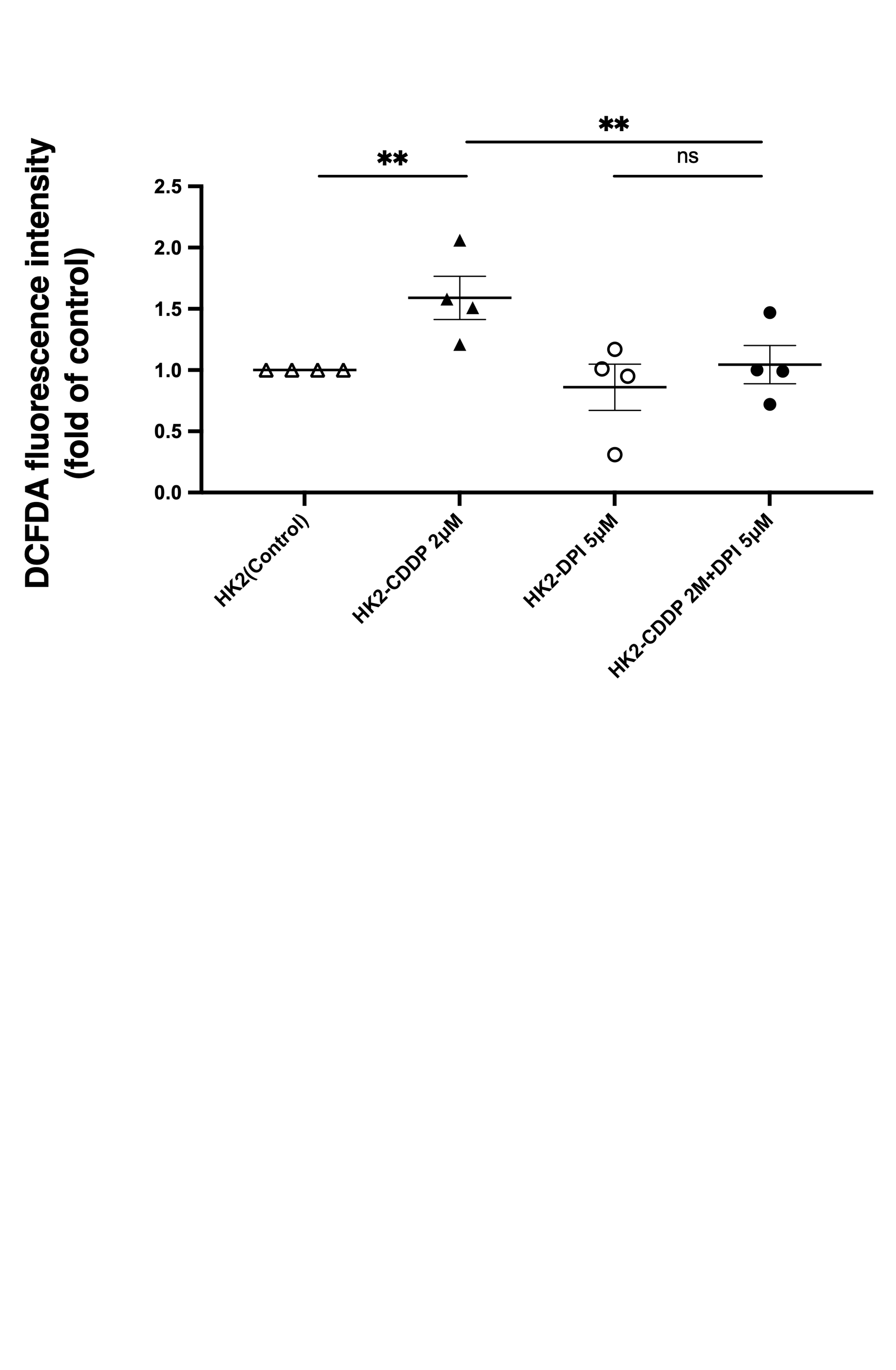

Supplement: SUPPLEMENTARY FIGURE 1 — Inhibition of ROS by DPI, NOX inhibitor, reduced cisplatin-treated human kidney tubular epithelial cells injury. ROS generation were a significant increase in cisplatin-treated HK2 cells which were inhibited by DPI. DCFDA is a fluorogenic dye that measures ROS activity within the cell which is detected by flow cytometry. Data represent the mean ± standard error of four data per group. [file Image_1.TIF]

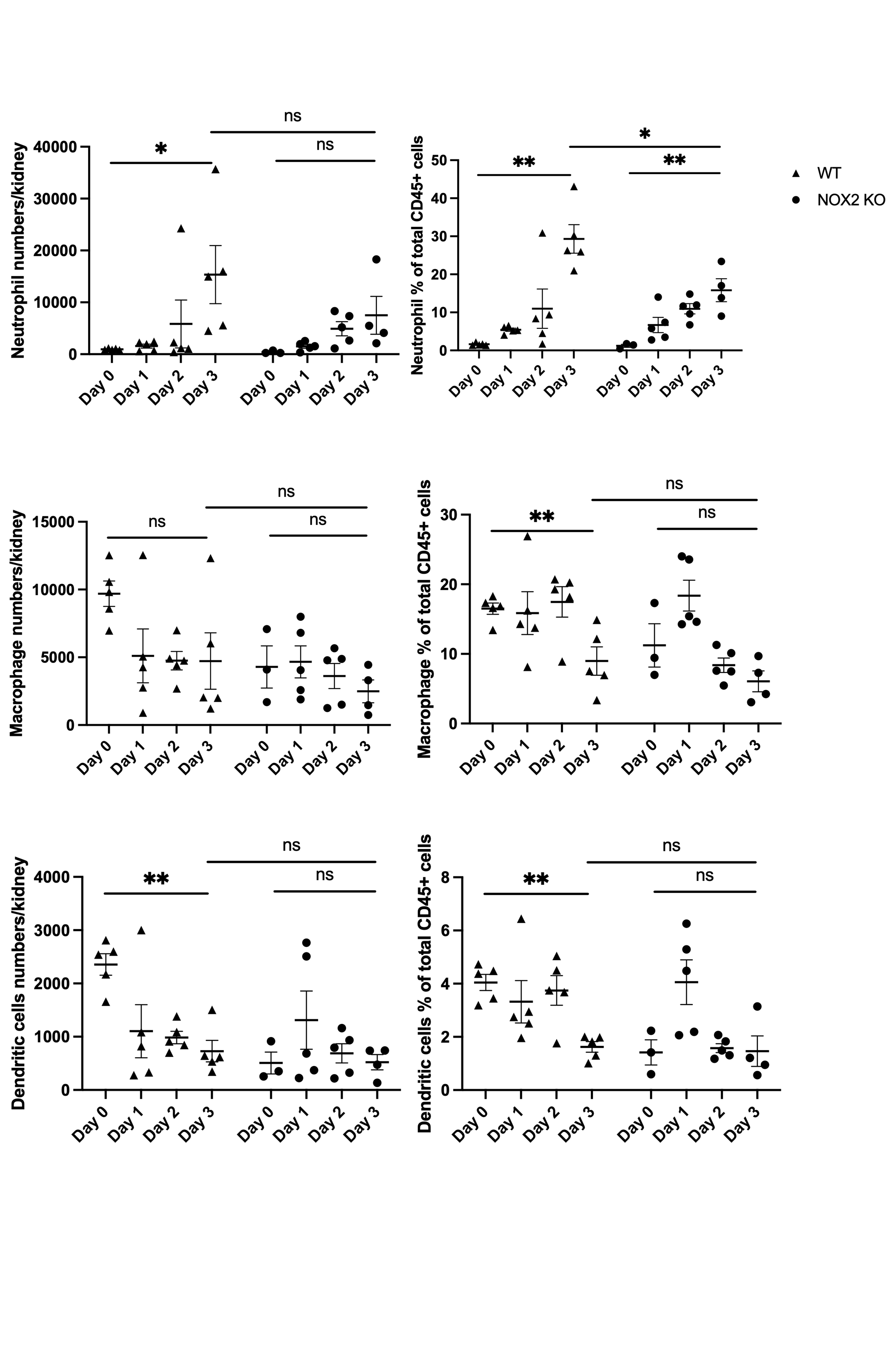

Supplement: SUPPLEMENTARY FIGURE 2 — Time course analysis neutrophil, macrophage, and dendritic cells from day 0 to day 3 of cisplatin injection in WT and NOX2 KO mice. [file Image_2.TIF]
